# Editorial: Endocytic and trafficking events in acute lung injury and pulmonary inflammation

**DOI:** 10.3389/fimmu.2024.1500369

**Published:** 2024-10-22

**Authors:** Laura A. Dada, István Vadász

**Affiliations:** ^1^ Divison of Pulmonary and Critical Care Medicine, Feinberg School of Medicine, Northwestern University, Chicago, IL, United States; ^2^ Department of Internal Medicine, Justus Liebig University, Universities of Giessen and Marburg Lung Center (UGMLC), German Center for Lung Research (DZL), Giessen, Germany; ^3^ The Cardio-Pulmonary Institute (CPI), Giessen, Germany; ^4^ Institute for Lung Health (ILH), Giessen, Germany

**Keywords:** endocytosis, intracellular trafficking, inflammation, acute respiratory distress syndrome, acute lung injury, influenza A virus, SARS-CoV-2, COVID-19

Lung inflammation and injury remain a major focus of research since the beginning of the coronavirus disease 2019 (COVID-19) pandemic. Various endocytic and intracellular trafficking events play a pivotal role in the integrity and physiological functions of the air-blood barrier but also in the invasion of pathogens, such as, e.g. respiratory viruses. Hence, disturbances in endocytosis and trafficking across and within alveolar epithelial and endothelial cells are important drivers of alveolar-capillary barrier dysfunction during respiratory inflammation and failure. Moreover, interfering with these endocytic/trafficking processes may serve as rescue measures in life-threatening sequels of lung damage in the context of acute respiratory distress syndrome (ARDS). Of note, several recent studies have focused on better understanding of lung cell-specific signaling patterns and endocytic events, utilizing novel technological advances in complex model systems. This Research Topic consists of original research manuscripts that enhance our knowledge on the mechanisms regulating the endocytic machinery during infection, inflammation and damage of the lung, as well as review articles that summarize most recent advances on the field.

The review article of Kryvenko and Vadász covers the six major endocytic pathways (1), namely clathrin-, caveolae-, endophilin- and glycosylphosphatidyl inositol-anchored protein-mediated endocytosis, as well as, macropinocytosis and phagocytosis and the subsequent trafficking events that are involved in alveolar-capillary barrier (dys)function during acute lung injury (ALI) and ARDS. In particular, the role of these internalization events in viral and bacterial infections of the respiratory tract and in the regulation of various transporters and junctional molecules during formation and clearance of protein-rich alveolar edema is described in detail. This manuscript also focuses on major technological advances, which have facilitated better understanding of intracellular trafficking events after endocytosis in various models and phases of ALI and describes potential novel therapeutic means, targeting these specific molecular events for patients with ARDS.

Most frequently, ARDS is a consequence of severe viral and/or bacterial pneumonia (2). Recently, the mechanisms of endocytosis of influenza A virus (IAV) and severe acute respiratory syndrome coronavirus 2 (SARS-CoV-2) by epithelial, and in particular by alveolar cells, have been intensively studied. An excellent review article in this Research Topic contributed by Hook and Bhattacharya covers important determinants of IAV deposition into the alveolar space, as well as the relevance of alveolar structure in the internalization process of IAV virions. The article also describes recent advances regarding the role of the alveolar glycocalyx and of the epithelial lining fluid (ELF) in IAV attachment and how post-endocytic events lead to failure of the alveolar-epithelial barrier. Clearly, further research will be necessary to assess the potential therapeutic role of interfering with cellular IAV attachment during initial infection and viral spread.

A key function of the alveolar-capillary barrier is providing adequate gas exchange that requires a relatively “dry” alveolar compartment where the epithelium is covered by a thin ELF layer (3). In contrast, during ARDS the alveolar space is flooded by protein-rich edema fluid, persistence of which is associated with worse outcomes (2). The research article of Alberro-Brage et al. describes a novel mechanism by which IAV in cultured epithelial cells and in three-dimensional precision cut lung slices impairs alveolar uptake and thus averts removal of excess protein content from the alveolar space *in vivo*. The authors establish the central involvement of matrix metalloprotease-14 as a negative regulator of megalin, a major receptor for albumin internalization in the alveolar epithelium (4, 5), which promotes shedding of the megalin ectodomain from the cell surface (6), thereby impairing its function. Therefore, inhibition of metalloprotease-14 may rescue alveolar protein clearance in the setting of ALI/ARDS.

In case of SARS-CoV-2, infection occurs when the spike protein of the virus that contains the receptor binding domain (RBD) binds to the host cell receptor angiotensin-converting enzyme 2 (ACE2) (7). Of note, patients with chronic lung diseases are more susceptible for cellular SARS-CoV-2 entry (8) in part as a consequence of elevated ACE2 expression levels (9). The original article of Chen et al. demonstrates that hypercapnia (an elevated level of CO_2_ in blood and tissues), which is often observed in patients with chronic lung diseases, increases ACE2 protein expression and viral infection in cultured human bronchial epithelial cells and in murine airway epithelium. Of note, similar effects were observed when bronchial epithelial cells were exposed to extracts of cigarette smoke, the most common cause of chronic lung disease. Moreover, both hypercapnia and cigarette smoke extract increased total cellular and lipid raft-associated cholesterol, which was found to be relevant for viral entry and assembly. Further to this notion, inhibition of cholesterol synthesis and depletion of cellular cholesterol reduced ACE2 expression and SARS-CoV-2 infection, revealing a novel and pharmacologically targetable mechanism that may contribute to the poor clinical outcomes of smokers and patients with advanced lung disease with hypercapnia upon COVID-19.

As compared to other etiologies of viral pneumonia, COVID-19 presents with higher levels of vasculopathy, which may lead to an array of complications including thrombotic events and pulmonary edema due to loss of alveolar-capillary barrier function (10, 11). Romero et al. characterized the pathways that are involved in SARS-CoV-2-triggered vasculopathy and identified a potential novel therapeutic strategy to ameliorate endothelial dysfunction during and post COVID-19. The authors found that activity of the epithelial sodium channel (ENaC), which is also expressed in the endothelium (12), is markedly inhibited by SARS-CoV-2 RBD. RBD also increased oxidative stress and production of tissue factor, causing barrier dysfunction and coagulopathy. Importantly, these effects could be restored by a TNF-derived peptide, called TIP that directly binds ENaC. TIP also limited pneumococcal infection, which was stimulated by SARS-CoV-2. Thus, TIP might represent a potent therapeutic mean, as it appears to stabilize/activate ENaC in both the alveolar endothelium and epithelium (13, 14) and may limit bacterial infections secondary to COVID-19.

Air-blood barrier integrity is also critically dependent on pulmonary surfactant (PS) that covers the alveolar epithelium, thereby reducing surface tension, and also plays an important role in innate immunity of the lung (15). PS is synthetized in the lamellar bodies (LB) of alveolar type 2 (AT2) cells and consists of phospholipids, cholesterol and four specific proteins, whereas some of the lipids that are not synthesized in the LB are transported to it via the Golgi apparatus (16, 17). While it is clear that PS alterations are associated with impaired barrier function (18, 19), the mechanisms that are at play remain less understood. Novel bioimaging techniques, described in detail in the review article by Garcia et al., which combine microscopy and spectroscopy, enable precise temporo-spatial depiction of LB structure and function during maturation, PS secretion and recycling in two- and three-dimensional cell culture systems. This is particularly remarkable, as better understanding of the implicated impairments e.g., during synthesis and transport to the alveolar surface may lead to therapeutic options in PS alterations associated with lung diseases.

During the inflammatory phase of ARDS and mechanical ventilation, an extracellular release of adenosine triphosphate (ATP) is observed that drives alveolar-capillary barrier dysfunction and thus, lung edema formation, in part through activation of purinergic, G-protein-coupled P2Y receptors (20, 21). In their manuscript, Kargarpour et al. convincingly demonstrate that in the setting of lipopolysaccharide (LPS)-induced ALI in mice, which triggers an acute recruitment of neutrophils to the lung; the purinergic P2Y2 receptors drive inflammation. Furthermore, neutralizing extracellular ATP, blocking P2Y2 and genetically deleting the receptor in neutrophils reduced neutrophil recruitment and rescued the inflammatory phenotype, which may open new avenues for therapy in the treatment of ARDS.

Endogenous glucocorticoids limit inflammation in ARDS, however systemic inflammation-associated glucocorticoid resistance may lessen these effects (22). Mahida et al. study the role of 11β-hydroxysteroid dehydrogenase type-1 (HSD-1), which converts inactive cortisone to active cortisol, in both alveolar macrophages in broncho-alveolar lavage samples from patients with sepsis-associated ARDS, in mice in LPS-induced ALI, as well as in a murine polymicrobial sepsis model (cecal ligation and puncture). The work establishes that HSD-1 activity of alveolar macrophages is markedly reduced in these scenarios, leading to decreased macrophage efferocytosis (phagocytosis of apoptotic cells), which may contribute to worse outcomes and thus, identifies HSD-1 as a potential therapeutic target in patients with sepsis and sepsis-associated ARDS.

Collectively, the articles published in this Research Topic highlight the pivotal role of alveolar-capillary endocytic and trafficking events in the pathogenesis and potential therapy of pulmonary inflammation and injury. Further studies to explore the therapeutic potential of these pathways are warranted.
